# Liver function during mechanical circulatory support: from witness to prognostic determinant

**DOI:** 10.1186/s13054-016-1312-7

**Published:** 2016-06-01

**Authors:** Christian Jung, Malte Kelm, Ralf Westenfeld

**Affiliations:** University Hospital Düsseldorf, Heinrich-Heine-University Düsseldorf, Medical Faculty, Division of Cardiology, Pulmonology and Vascular Medicine, Moorenstrasse 5, D-40225 Düsseldorf, Germany

## Abstract

In recent years, the treatment options for patients with severe cardiorespiratory failure have been extended by the implementation of mechanical circulatory support (MCS). Identification of patients that benefit most from this cost-intensive treatment modality is of central importance, but is also challenging. Previous studies unravelled certain patient characteristics that should be taken into account, such as age, weight, and underlying pathology, and also the delay until MCS implementation as well as tissue hypoxia as prognostic factors. Relevant comorbidities included neurologic, renal, and hepatic disorders. Of note, baseline liver function tests predicted outcome in patients on extracorporeal life support (ECLS), including short-term and long-term mortality. Most strikingly, increased levels of alkaline phosphatase and total bilirubin indicated unfavourable short-term and long-term survival even after adjustment for age, gender, left ventricular function, and relevant known comorbidities such as impaired renal function and diabetes. Therefore, the assessment of liver function tests may be regarded as another piece in the complex puzzle of our efforts perceiving the ideal ECLS candidate with positive long-term outcome.

Over the last decade, the treatment of patients with severe cardiorespiratory failure has witnessed a dynamic evolution of new treatment options [1, 2]. This includes providing temporary support for cardiac or pulmonary function using mechanical circulatory support (MCS) devices, referred to as extracorporeal life support (ECLS). Available devices provide adequate oxygenation and cardiac output whilst bypassing the physiological circulation, and are usually implemented in the emergency department or on intensive care units. Treatment protocols for peripheral veno-arterial implementation in patients with cardiogenic shock or as escalation during cardiopulmonary resuscitation have changed the landscape of therapeutic options in this patient population [3, 4]. In addition, using ECLS provides a temporary option to bridge patients towards recovery or alternative therapies such as heart transplantation or durable assist devices. However, in the light of the increasing number of MCS implementations, identifying patients that benefit from these cost-intensive treatment modalities is of utmost importance to facilitate individual decision-making based on a thorough risk–benefit evaluation. Although cardiocirculatory failure is often the central underlying aetiology, the prognosis of these patients is often determined by (multi-)organ failure [5]. Therefore, the article by Roth et al., investigating whether liver function predicts outcome in patients on ECLS, addresses an important topic [1].

The central dilemma of the quick decision as to who should or should not receive ECLS therapy is the lack of data. No randomised trials exist, and thus available data are mainly derived from retrospective registries or anecdotal cases. Still, there is agreement that contraindications to ECLS present conditions associated with particularly poor outcome. These have been recently reviewed in this journal [6]. Poor ECLS candidates are patients with severe neurologic injuries, intracranial or major haemorrhage, immunosuppression, (irreversible) multi-organ failure, disseminated malignancy, or patients with advanced age or already impaired quality-of-life. In addition, ECLS is not possible in patients with aortic dissection or severe aortic regurgitation since this leads to further deterioration of the underlying pathology. Another consideration that needs to be taken into account is that treatment modalities have to be available after successful stabilization with ECLS, such as heart transplantation or ventricular assist device therapy in case of persisting heart failure.

Besides some smaller registries indicating that the duration [7] and extent of tissue hypoxia as determined by increased lactate levels [4, 5] have prognostic implications, the most advanced database has been published by Schmidt et al. leading to the construction of the “survival after veno-arterial-ECMO” (SAVE) score [8]. Along with selected patient characteristics, such as age, weight, underlying pathology, and also delay of MCS implementation and tissue hypoxia, the likelihood of survival can be calculated. Relevant comorbidities include neurologic, renal, and hepatic disorders.

In their article, Roth et al. confirm that, in patients undergoing veno-arterial ECLS therapy following cardiovascular surgery, liver function tests are strong predictors of short-term and long-term mortality. The majority of the patients suffered from circulatory failure or cardiogenic shock defined as hypotension despite optimal fluid and catecholamine therapy and signs of anaerobic metabolism. Although this cohort of postoperative patients differs from patients that receive ECLS support in more urgent situations, the study indicates the usefulness of evaluating liver function before ECLS initiation. Increased levels of alkaline phosphatase and total bilirubin indicated increased short-term and long-term mortality even after adjustment for age, gender, left ventricular function, and relevant known comorbidities such as impaired renal function and diabetes. The authors conclude that these values could possibly help guide therapy decisions.

These data are in line with recent findings stressing the importance of liver function and dysfunction in cardiovascular patients. This applies for different patient cohorts. Murata et al. provided convincing evidence that the assessment of liver dysfunction using the model of end-stage liver disease (MELD) scores can be used for predicting postoperative morbidity and mortality in patients undergoing cardiac surgery [9]. In parallel, the assessment of liver function also provides prognostic information in patients with heart failure [10] or patients receiving heart transplantation [11]. Other laboratory values are also of interest for evaluating liver function in cardiovascular diseases. Increased international normalized ratio (INR) has been proven to be an independent predictor of all-cause mortality in acutely decompensated heart failure patients without anticoagulation, summarizing coagulation abnormalities and hepatic insufficiency [12]. Okada and co-workers speculated that their finding in heart failure patients could be related to systemic inflammation, neurohormonal activation, or venous congestion which might also differ within this patient population [12].

Another publication sheds additional light on the prognostic role of the liver in patients treated with ECLS. Mazzeffi et al. were able to show that 8 % of ECLS patients develop acute liver failure during ECLS, while patients with chronic liver disease or acute liver failure prior to ECLS initiation were excluded. In this study, the median ECLS duration for developing acute liver failure was 5 days and these patients has distinctly elevated mortality rates [13].

These recent findings stress the clinical relevance of inter-organ crosstalk mainly either on the basis of haemodynamic interplay or due to humoral soluble factors. Furthermore, these results deem the treating physician to take hepato-cardiac comorbidities into account during risk stratification and therapy planning. The findings of Roth et al. may be regarded as another piece in the complex puzzle of our efforts perceiving the ideal ECLS candidate with favourable long-term outcome.

## Abbreviations

ECLS: extracorporeal life support
MCS: mechanical circulatory support
